# Scottish survey of diabetes services for minority ethnic groups

**DOI:** 10.1186/1472-6963-6-130

**Published:** 2006-10-09

**Authors:** Hamid R Baradaran, Joan Jamieson, Rafik Gardee, Robin P Knill-Jones

**Affiliations:** 1 Public Health and Health Policy Section, University of Glasgow, Glasgow G12 8RZ, UK; 2 Information Services Division c/o Health Scotland, Clifton House. Glasgow G3 7LS, UK

## Abstract

**Background:**

In the UK, all ethnic minority groups have higher rates of diabetes than the general population. Although there have been a number of projects to assess diabetic care amongst minority ethnic groups in the United Kingdom, little is known about the extent to which the needs of ethnic minority groups are actually met by the National Health Service (NHS) Scotland. Therefore we conducted this study to understand of the current situation for diabetes care available to minority ethnic groups in Scotland.

**Methods:**

We conducted this cross-sectional study in all health boards in Scotland. A questionnaire was designed based on expert comments. It was completed by Local Health Care Cooperatives (LHCC) managers, chairs, diabetes specialist nurses and public health practitioners.

**Results:**

57 of questionnaires were returned (response rate = 69.5%). Of these LHCCs, 71% responded that diabetes was part of their LHCC plan. However 69% answered that ethnic group was not recorded by community services and GPs, and 80% of LHCCs did not monitor trends of complications of diabetes by ethnic group.

**Conclusion:**

Improvement is needed in quality, completeness, and availability of minority ethnic group data for diabetes at a national level, particularly if NHS Primary Care Organisations are to be responsible for providing diabetes care as laid out in the Scottish Diabetes Framework.

## Background

The prevalence of diabetes worldwide varies from approximately 2 to 50% within different ethnic groups [1,2]. In the UK, all ethnic minority groups have higher rates of diabetes than the general population. Studies in the United Kingdom have shown diabetes prevalence rates of 11–20% in Asian Indians, 15% in Afro-Caribbeans and 1–5% in White Caucasians. Generally type 2 diabetes is up to four times more common in British South Asians than in the indigenous White population [3-11]. During the past decades the UK has become an increasingly multicultural society with the settlement of immigrants and refugees together with their families from the Middle East, Africa, Asia, and Eastern Europe. For instance, minority ethnic groups comprise 2% (101,677 residents) of the total population in Scotland. Table 1 describes the Scottish population by NHS Board based on the 2001 Census. One policy of the Scottish Executive Health Department (SEHD) aims to offer equal access to medical care for all residents, regardless of social class or ethnicity [12]. The NHS in Scotland consists of 15 Health Boards. Each Board has a Primary Care Trust (PCT) and smaller Local Health Care Cooperatives (LHCCs). The role of the LHCC is to find out what needs to be done locally to improve their population's health and health care services, and then plan and develop primary care services to meet these needs. Although there have been a number of projects to assess diabetes care amongst minority ethnic groups in the UK, little is known about the extent to which the needs of ethnic minority groups are actually met in Scotland. This study carried out to understand the situation in the LHCCs with regard to the provision of diabetes care to minority ethnic groups.

## Methods

Because there was no specific tool to assess the current situation for diabetes care generally and in particular for ethnic minority groups in Scotland, a questionnaire needed to be designed. Therefore we gathered ideas and comments from experts in this field. The first version of the questionnaire was designed, then it was sent to three LHCCs where Managers, Chairs, Diabetes Specialist Nurses and Public Health Practitioners completed and made comments. One of the recommended additions was to have some questions about availability of Scottish Intercollegiate Guidelines Network for Diabetes (SIGN Guideline 55) in the final version of the questionnaire. The questionnaire as distributed consisted of twelve closed questions in addition an open question. A total of 82 questionnaires were posted out to all LHCC managers in Scotland in March 2003 with a covering letter requesting completion of the questionnaire and an addressed envelope for return. Two weeks later, this was followed up with a reminder letter.

## Results

57 (69.5%) questionnaires were received representing: 22 urban, 16 rural and 19 mixed rural and urban setting (small towns and rural) LHCCs. One LHCC provided a development plan. Non-respondents were 25 LHCCs representing: 10 urban, 7 rural and 8 mixed rural and urban setting (small towns and rural). A total of 28 comments were submitted by six LHCCs. Their comments were summarised into: Issues related to collecting information about ethnic group (N = 6); Size of minority ethnic groups (N = 4); Information systems' inability to collect and store ethnic data (N = 8); Use of Interpreters (N = 3); Training of staff (N = 3); Health promotion materials (N = 4). The Table 2 describes the overall summary of the twelve questions within the questionnaire.

## Discussion

The findings of the present study indicate that recording of ethnic group has not been considered a priority by LHCCs despite the importance of ethnicity in determining prevalence and complications of diabetes.

### Census

Of the LHCCs 66% did not collect data based on the 2001 census Ethnic Categories. Without this basic information, it is difficult for LHCCs to even begin to identify their demographic profile in order to assess the health needs of their minority ethnic population, although a demographic profile is a basic requirement of providing culturally sensitive and competent services.

### GPs records & SIGN-55

The high response of GP practices and community services indicating that they do not record ethnic group (69%) can, in part, be attributed to current information systems. However, there seems to be some inconsistencies in respondents' responses, as 58% state that SIGN-55 guidelines [13], which indicates ethnic group as one of the core data items, is recorded on GP systems, but appears not to be acted upon. It is no surprise, therefore, that 80% of respondents report their inability to combine ethnic demographic data with other data sources to plan services and monitor complication trends between ethnic groups. Having such information would seem to be an important starting point in the ability of NHS organisations to reach a position to target their finite resources more effectively. The absence of robust information is disconcerting, in that diabetes is reported to be specifically identified by 71% of LHCC plans. Only one LHCC supplied their LHCC plan, describing their demographic profile by ethnic group and their intention to target more effectively Minority Ethnic Groups by joint working with a neighbouring LHCC and involving leaders of local minority ethnic health group.

From the comments section, there is also evidence that altering attitudes of NHS organisations requires ongoing change management, education and training. In some LHCCs numbers appear to be small as some respondents concluded: "...*As percentage very low (referring to Minority Ethnic Groups) there is no specific section within the LHCC plan. All patients are treated equally within the LHCC*," and another: "*Numbers are so small we do not have a specific programme for ethnic groups*," and another: "*As patient records are confidential don't know what SIGN-55 data is recorded*."

### Cultural competency

The findings that only 55% of LHCCs have access to interpreters, 55% do not record cultural/religious requirements, 24% have no culturally appropriate dietetic counselling and 33% have no appropriate health information materials available suggests that much is still required in improving the patient journey for ethnic minorities within the NHS.

Training and development of NHS staff are needed to help bring about such radical service improvements, giving people at the frontline the opportunity to develop appropriate skills and resources to do a better job [14]. This builds on Our National Health [14], which gave a commitment to: "...*Ensure that NHS staff are professionally and culturally-equipped to meet the distinctive needs of people and family groups from minority ethnic communities*". Therefore, it is concerning that 42% of respondents report that their LHCC staffs are not trained in diabetes in relation to Minority Ethnic Groups and 13% are unsure. Patients normally have their first contact with Primary Care staff, who manage 90% of patient contacts within the NHS.

### Limitation of the study

Similar to all research, this study had limitation. Since there was lack of census data on the number of ethnic minorities in each LHCC, therefore we could not compare and analyse the differences between LHCCs.

## Conclusion

This study indicates that cultural health care practices should be improved and staff attitudes changed by the delivery and uptake of effective and appropriate training, and as part of this effort community participation by minority ethnic groups should be encouraged to assist in identifying their specific needs for targeted health services and related information.

## Competing interests

The author(s) declare that they have no competing interests.

## Authors' contributions

JJ and HRB developed questionnaires and performed the data collection. HRB analysed the data and wrote the first manuscript. RG and RPKJ participated in the development of study design, editing and revising the manuscript. All authors contributed to and have read and approved the final manuscript.

## Pre-publication history

The pre-publication history for this paper can be accessed here:



## Figures and Tables

**Table 1:** The demographics of health boards in Scotland

**Health Board**	**All people**	**White**	**Indian**	**Pakistani and other South Asian**	**Chinese**	**Other**
**Argyll & Clyde**	420491	416347	775	1040	801	1528
**Ayrshire & Arran**	368149	365640	489	459	670	891
**Borders**	106764	106175	73	106	103	307
**Dumfries & Galloway**	147765	146796	118	174	244	433
**Fife**	349429	345003	514	1470	750	1692
**Forth Valley**	279480	276300	344	1286	455	1095
**Grampian**	525936	517392	1074	1586	1627	4257
**Greater Glasgow**	867150	827832	6564	20676	4912	7166
**Highland**	208914	207243	165	388	271	847
**Lanarkshire**	552819	546260	911	2756	1202	1690
**Lothian**	778367	756584	2713	7094	3974	8002
**Orkney**	19245	19159	17	7	17	45
**Shetland**	21988	21756	19	59	27	127
**Tayside**	389012	381517	1244	2816	1243	2192
**Western Isles**	26502	26330	17	53	14	88

**Table 2:** The results of the Questionnaire (Responses by Percentage)

**Question**	**Yes (%)**	**No (%)**	**Not sure/No response (%)**	**In progress (%)**
Can you describe your LHCC by Census 2001 Ethnic Categories	22	53	12	13
Do Community Services & GPs record ethnic group	7	69	15	9
Do you combine ethnic demographic data with other sources to plan services	2	80	13	5
Is Diabetes part of your LHCC plan	71	11	7	11
Does your LHCC collect SIGN 55 data	58	9	21	11
Is SIGN 55 data recorded on GP systems	36	15	28	22
Does the LHCC monitor trends of complications by ethnic group	2	80	17	2
Are LHCC staff trained in Diabetes related to BMEGs	27	42	20	11
Do all patients have access to interpreters	55	20	18	7
Are procedures in place to record cultural/religious requirements	9	55	37	0
Is there appropriate dietetic counselling	35	24	34	7
Are appropriate health information materials available	29	33	31	7
